# Spectrum of Gastroenteropancreatic NENs in Routine Histological Examinations of Bioptic and Surgical Specimen: A Study of 161 Cases Collected from 17 Departments of Pathology in the Czech Republic

**DOI:** 10.1155/2014/373828

**Published:** 2014-02-18

**Authors:** Václav Mandys, Tomáš Jirásek

**Affiliations:** Department of Pathology, Third Faculty of Medicine, Charles University and University Hospital King's Vineyards, Srobarova 50, 100 34 Prague 10, Czech Republic

## Abstract

*Objective.* To characterize GEP-NENs in routine biopsies and surgical specimen in the Czech Republic and to evaluate how WHO Classification (2010) is acceptable in diagnostic practice. *Methods.* Paraffin-embedded blocks and bioptic reports were collected from 17 departments of pathology. Histologic slides were stained with H&E and immunohistologically for CgA, synaptophysin, and Ki-67. *Results.* Out of 28 gastric NENs, there were 22 NETs, G1, 5 NETs, G2, and 1 NEC. Ten duodenal NENs were NETs, G1. Among 27 NENs of jejunum and ileum, 23 were NETs, G1, 2 NETs, G2, and 1 NEC and 1 mixed adenoneuroendocrine carcinoma (MANEC). Among 42 appendiceal “incidentalomas”, 39 were NETs G1, 2 goblet cell carcinoids, and 1 MANEC. Out of 34 large intestinal NENs, 30 were NETs, G1, 3 NETs, G2, and 1 NEC. One small intestinal and 6 large bowel neoplasms were reclassified as poorly differentiated adenocarcinomas. In 12 pancreatic NENs, there were 7 NETs, G1, 3 NETs, G2, and 2 NECs. *Conclusions.* Our study demonstrates differences in GEP-NENs frequency in sites of origin in our region, comparing to other countries. Regarding routine bioptic diagnostics, we gave evidence that the WHO 2010 classification of NENs is fully acceptable for exact categorisation of tumours.

## 1. Introduction

Gastroenteropancreatic neuroendocrine neoplasms (GEP-NENs) represent a group of tumours of increased clinical significance [1, 2]. Recently, updated guidelines for the management of patients with digestive neuroendocrine tumours and current diagnostic procedure have been published [3, 4]. One of the essential components of the diagnostic protocol is bioptic examination that enables establishing histopathologic typing, grading, and staging of individual tumours [5]. A standardized pathology report is the need for adequate management of patients [6]. Frequency of GEP-NENs, their appearance within the GIT, and the spectrum of their histologic types are to some extent different in individual countries [7–14]. The aim of this study was to collect the cases of GEP-NENs from different departments of pathology in the Czech Republic, characterize the spectrum of GEP-NENs diagnosed in these departments in two-year period from 2007/01/01 to 2008/12/31, analyse the data obtained from bioptic reports, and evaluate how newly formulated WHO classification (2010) [15] is acceptable in regular (common) diagnostic practice.

## 2. Materials and Methods

All tumours diagnosed as carcinoids, neuroendocrine tumours, or neuroendocrine carcinomas in two-year period from 2007/01/01 to 2008/12/31 in 17 different departments of pathology represented by 5 university hospitals and 12 regional hospitals and laboratories were included into the study. These departments were randomly selected from total of 96 departments of pathology within the whole Czech Republic. The day of inclusion was the day of pathological report.  Paraffin-embedded tissue blocks and bioptic reports from participating departments were collected at the Department of Pathology, Third Faculty of Medicine, Charles University and University Hospital King's Vineyards, Prague, Czech Republic. Five-micron-thick paraffin sections were prepared from all collected blocks and stained with routine hematoxylin and eosin and by immunohistochemistry to detect chromogranin A (CgA), synaptophysin, and Ki-67. All antibodies used for immunohistochemistry were purchased from DakoCytomation (Glostrup, Denmark). Polyclonal rabbit anti-CgA was diluted 1 : 600, monoclonal anti-synaptophysin (clone SY 38) was diluted 1 : 20, and monoclonal anti-Ki-67 (clone MIB-1) was diluted 1 : 50. Histofine detection system (Exbio, Czech Republic) and 3-3′ diaminobenzidine as a chromogen were used to visualize immunohistologic reaction. The slides were evaluated independently by two pathologists specialized in NEN diagnostics (Václav Mandys and Tomáš Jirásek) under an optical microscope. Presented diagnostic conclusions are based on consequent consensus.

## 3. Results 

Overall information on the number of tumours in individual sites, age of patients, and source of tissue samples are summarized in Table 1.

Histologic classification of tumours is summarized in Table 2.

**Figure 1:**
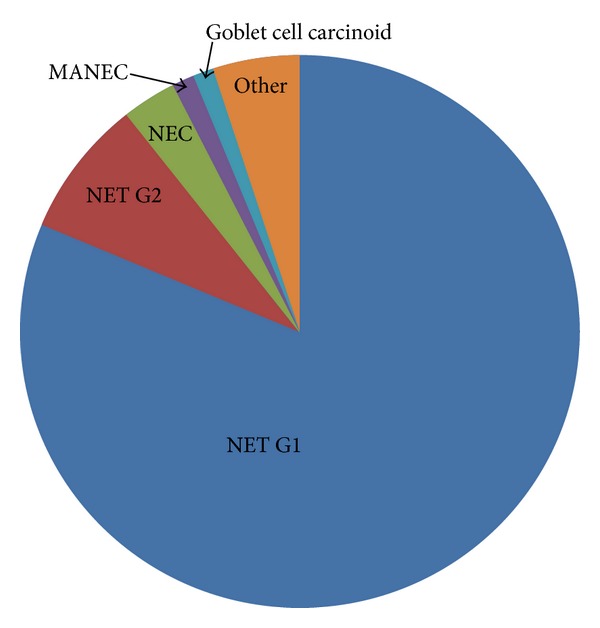
Distribution of GEP-NENs according to histologic type of tumour. NET, neuroendocrine tumour; NEC, neuroendocrine carcinoma; MANEC, mixed adenoneuroendocrine carcinoma.

Distribution of tumours according to histologic type is introduced in Figure 1.

### 3.1. Stomach

In 29 cases from stomach, endoscopic biopsies predominated (26 blocks). In 2 cases only, tissue blocks were obtained from surgical resection. Neuroendocrine tumours (NETs) predominated in this group (27 cases). In one case only the tumour was classified as neuroendocrine carcinoma (small cell carcinoma). In one case the tumour was reclassified as nonneuroendocrine carcinoma, probably metastatic, from anamnestically preexisting breast carcinoma of nonspecified histologic type. Detection of CgA and synaptophysin was positive in all NETs in most cells. In the case of small cell carcinoma, immunoreactivity for CgA and synaptophysin was evident only focally. In the tumour finally diagnosed as metastatic carcinoma, immunodetection of CgA was negative and synaptophysin was detected small number of tumour cells only. Grading of NENs was as follows: NET G1 22 cases, NET G2 5 cases, NEC G3 1 case. Other changes observed in evaluated slides were represented by chronic atrophic gastritis (19 cases). In 1 patient the history of metachronous gastric carcinoma was noted.

### 3.2. Duodenum

In 10 patients with duodenal tumours predominated endoscopic biopsies (8 cases), 2 patients underwent resection. All tumours were classified as NETs G1. All tumours showed strong immunostaining for CgA and synaptophysin in the majority of neoplastic cells and displayed very low proliferative activity corresponding with G1 (Ki-67 index ≤2). In one case, evaluation of Ki-67 index was difficult due to limited extent of tissue samples. In one case anamnestic data on synchronous gastrointestinal stromal tumour (GIST) in the small intestine were introduced in the bioptic report.

### 3.3. Jejunum and Ileum

In 20 patients (out of all 28 cases), the primary site of tumour was ileum, in 1 patient jejunum, and in remaining 7 cases the site was introduced as small intestine, not otherwise specified. Resection specimens were obtained from 24 patients; endoscopic biopsies were in 4 cases only. Tumours were classified as NET G1 in 23 cases and NET G2 in 2 cases. One neoplasm was classified as NEC G3, a small cell type and one case as mixed adenoneuroendocrine carcinoma (MANEC). Remaining tumour was re-classified as poorly differentiated adenocarcinoma.

In all NETs strong immunoreactivity for CgA and synaptophysin was detected in the vast majority of cells. In 3 cases, diagnosis of carcinoma was established. One of them was small cell NEC, with only focal expression of CgA and synaptophysin and with high proliferative activity of tumour cells (Ki-67 index exceeding 50%), corresponding with G3. One case was diagnosed as MANEC, expressing CgA and synaptophysin, tumour grade G3. The last case, originally diagnosed as “carcinoid” was re-classified as poorly differentiated adenocarcinoma, without signs of neuroendocrine differentiation.

### 3.4. Appendix

All 42 appendiceal tumours were “incidentalomas”. Appendectomy was performed predominantly due to appendicitis (37 cases). In 2 cases, appendectomy was performed during the surgery for colorectal adenocarcinoma, in 1 case for ovarian carcinoma, in 1 case for cervical carcinoma, and in 1 case for endometrial carcinoma. In all cases, resection specimen was evaluated. NETs G1 predominated in this group (39 cases). Goblet cell carcinoid was diagnosed in 2 cases with predominating neoplastic infiltration of submucosa in one of them and with more extensive infiltration of tunica muscularis in the second tumour. Remaining one case was diagnosed as MANEC with broad neoplastic infiltration of tunica muscularis. All NETs displayed strong immunoreactivity for CgA and synaptophysin in almost all neoplastic cells. In all cases of goblet cell carcinoid/MANEC, CgA and synaptophysin were present only in scattered neoplastic cells. Ki-67 index was ≤2 in all NETs and up to 3% in goblet cell carcinoids. In MANEC, the Ki-67 index exceeded 30%.

### 3.5. Large Intestine

Out of 40 tumours located in the large intestine, 21 cases were in rectum, 1 in sigmoid colon, 2 in caecum, and 1 in ascending colon, and in 15 remaining cases, exact location of a tumour was not introduced in the bioptic report. In 30 patients endoscopic biopsies were available, and resection specimens were evaluated in 10 cases. NET G1 was classified in 30 cases and there were 3 NETs G2; one case was classified as NEC, small cell type. Remaining 6 tumours were re-classified as poorly differentiated adenocarcinomas. In one patient with NET G1, two synchronous colonic adenocarcinomas were described within the resection specimen. In all cases of NETs, strong expression of CgA and synaptophysin was detected. In the case of small cell carcinoma, immunohistological detection of CgA was negative, synaptophysin was focally positive and proliferative activity of tumour cells (Ki-67 index) was higher than 80%. In remaining 6 cases, tumours were re-classified as poorly differentiated adenocarcinomas. These tumours were either completely negative for neuroendocrine markers, or showed markers of neuroendocrine differentiation in scattered neoplastic cells only.

### 3.6. Pancreas

Resection specimens were available in all 12 cases. No anatomical sublocation of the tumour within the pancreas was introduced in all these tumours. Previous endoscopic biopsy in one patient with NET G1 was also available. Seven tumours were classified as NETs G1, and 3 tumours were NETs G2. Two tumours were neuroendocrine carcinomas (NEC), one of them of small cell type and the second one a large cell NEC. All G1/G2 tumours displayed strong immunoreactivity for CgA and synaptophysin. Both NECs were immunohistologically positive for synaptophysin, while immunodetection of chromogranin-A was negative in a small cell tumour. Proliferative activity (Ki-67 index) corresponded with tumour grades.

## 4. Discussion

Extended knowledge and increased clinical interest in neuroendocrine neoplasms urged the effort to formulate a new classification of these tumours. Definitions of individual tumour types introduced in the WHO Classification published in 2000 [16] and consequently refined especially by ENETS [9, 17–20] represented a new approach to classify NENs using not only pure histomorphologic criteria, but also clinicopathological correlation. Distinction of well-differentiated neuroendocrine tumour classified as potentially malignant from well-differentiated neuroendocrine carcinoma assessed as low-grade malignant was not based on the histological appearance of the tumour, but biologic behaviour of the tumour was estimated by the tumour size and extension. Despite of the progress, such approach appeared to some extent problematic in cases where endoscopic biopsies were available only.

Recently, a new WHO classification of tumours of the digestive tract (2010) introduces simplified categorisation of NENs [15]. The most substantial advantage of this new classification is definition of 4 essential tumour types, based on histomorphologic appearance and proliferative activity of tumour cells (Ki-67 index). Clear definition of these tumour categories (NET G1, NET G2, NEC, and MANEC) in all locations of the digestive tract eliminates diagnostic problems in endoscopically obtained small tissue samples.

The major change in the new WHO classification (2010) [15] is a new approach in coding of NENs according to ICD-O3. All 4 essential tumour types are coded in all locations as malignant (/3), with two exceptions only. This approach significantly differs from the WHO classification 2000 [16], where tumours of small size, low proliferative and mitotic activity, and displaying limited progression were classified as tumours of “benign” or “uncertain” behaviour and coded according to ICD-O3 as tumours of uncertain behaviour (/1).

Data on incidence of NEN and on their frequency in individual sites of origin within the GIT have been published in more studies. However, some of these data are to some extent controversial. Appendix as the most frequent site of NENs was observed not only in our study, but also in identical finding published by others [9, 14]. High frequency of NENs has also been described in the colon and rectum [10–12] and in the small intestine [7, 8]. More exceptionally, stomach has been mentioned as the main site of GEP-NENs [13]. Relatively higher number of appendiceal incidentalomas in our study could be explained by a fact that detailed bioptic examination of appendices has been still mandatory in the Czech Republic.

This study cannot contribute to evaluation of incidence of GEP-NENs in the Czech Republic. The data from Central Oncological Registry of the Czech Republic indicate the incidence approximately 4/100 000/year.

From the clinical point of view and to select the adequate therapeutic strategy it is necessary to estimate the risks of aggressive behaviour of the particular tumour. Apart from the tumour type, grade, and stage, location of the tumour plays an important role in this estimation. The need to classify the particular tumour precisely is evident. Our study demonstrates that before introduction of WHO 2010 classification the majority of tumours in original bioptic reports were classified as “carcinoids” (19 of 29 in stomach; 6 of 10 in duodenum; 14 of 28 in the small intestine; 23 of 42 in appendix; 23 of 40 in the large bowel). Approximately 1/3 of GIT tumours (52) and 11 of 12 pancreatic tumours were classified in accordance with the WHO 2000 Classification as well-differentiated neuroendocrine tumours or well-differentiated neuroendocrine carcinomas. Major diagnostic discordancy was found in 10 cases. Six of these tumours represented undifferentiated carcinomas of the large bowel which were originally diagnosed 3x as MANEC, 2x as malignant carcinoid, and 1x as neuroendocrine carcinoma. One poorly differentiated carcinoma of jejunum was originally diagnosed as carcinoid. Small cell carcinomas, 1 in stomach and 1 in ileum, were originally classified as malignant carcinoid and carcinoid, respectively. One gastric tumour originally diagnosed as carcinoid was re-classified as non-neuroendocrine carcinoma, probably metastasis of breast cancer.

In our opinion, diagnostic process of NENs should be performed in close cooperation with the clinicians, especially oncologists. Biphasic diagnostic procedure of NENs is recommended in our conditions (the Czech Republic). The first (essential) diagnosis of a NEN is performed in all departments of pathology. Consequently, usually based on the clinician request, extended and more detailed diagnosis, including detection of wider spectrum of tumour markers, like hormonal products, is performed in specialized centres by the “second opinion”. Such approach contributes to improvement of diagnostic procedure especially in rare challenging cases and in patients with unusual clinical symptomatology.

## 5. Conclusions

The results of our study demonstrate the frequency of individual types of GEP-NENs in routine bioptic examinations and appearance of NENs in different sites of origin within the GIT. We demonstrate slight differences in primary sites of GEP-NENs in our region, comparing to other countries. Relatively higher number of appendiceal incidentalomas in our study could be explained by a fact that detailed bioptic examination of appendices has been still mandatory in the Czech Republic. Our study gave evidence that the WHO 2010 classification of NENs represents fully acceptable approach for histopathologic diagnosis and enables categorising the tumours without difficulties.

## Figures and Tables

**Table 1:** Number of tumours in individual sites, age of patients, and source of tissue samples.

Site	Number of patients	Male : female ratio	Age: years (range)	Resection	Biopsy
Stomach	29	11 : 18	66 (41–85)	2	27
Duodenum	10	6 : 4	59 (27–76)	2	8
Jejunum and ileum	28	16 : 12	65 (37–94)	24	4
Appendix	42	11 : 31	38 (10–85)	42	0
Large intestine and rectum	40	28 : 12	42 (29–84)	10	30
Pancreas	12	4 : 8	66 (44–79)	11	1

Total	161	76 : 85	56 (10–94)	91	70

**Table 2:** Histologic classification of tumours with their number in individual sites.

Site	NET G1	NET G2	NEC	MANEC	Goblet cell carcinoid	Other	Total
Stomach	22	5	1	0	0	1	29
Duodenum	10	0	0	0	0	0	10
Jejunum and ileum	23	2	1	1	0	1	28
Appendix	39	0	0	1	2	0	42
Large intestine and rectum	30	3	1	0	0	6	40
Pancreas	7	3	2	0	0	0	12

Total	131	13	5	2	2	8	161
%	81.37	8.07	3.11	1.24	1.24	4.97	100
